# Impact of Underlying Comorbidities on Mortality in SARS-COV-2 Infected Cancer Patients: A Systematic Review and Meta-Analysis

**DOI:** 10.31557/APJCP.2021.22.5.1333

**Published:** 2021-05

**Authors:** Harmanjeet Kaur, JS Thakur, Ronika Paika, Shailesh M Advani

**Affiliations:** 1 *Department of Community Medicine & School of Public Health, PGIMER, Chandigarh, India. *; 2 *Cancer Prevention and Control Program, Georgetown University School of Medicine, Georgetown University, Washington DC, USA. *

**Keywords:** COVID-19, carcinoma, prevalence, case fatality, review

## Abstract

**Background:**

The evidence has shown that SARS CoV-2 infected patients with comorbidities are more likely to have severe disease sequel and mortality. In SARS-CoV-2 infected cancer patients risks associated with other underlying comorbidities might vary from those in non-cancer SARS CoV-2 infected patients. The relative impact of different underlying health conditions among patients with cancer and SARS CoV-2 infection remains yet to be explored. This systematic review aims to explore the prevalence of comorbidities among cancer patients with SARS CoV-2 infection and their impact on mortality.

**Methods:**

Online databases PubMed, Embase, Scopus and Web of science were searched for articles published between 9th July 2019 to July 8th 2020.Studies of cancer patients (>18 years) with diagnosis of SARS CoV-2 infection, published in English were included. A random-effects modelling for the meta-analyses was applied to assess the pooled prevalence and odds ratio for mortality due to comorbidities in SARS CoV-2 infected cancer patients.

**Results:**

Total 31studies with 4086 SARS-CoV-2 infectedcancer patientsmet the inclusion criteria. Most prevalent co-morbidities in cancer patients with SARS CoV-2 infection were hypertension [42.3% (95%CI:37.5- 47.0)], diabetes [17.8% (95% CI: 15.3-20.4)] and cardiovascular diseases [16.7% (95%CI:12.9-20.4)].The risk of mortality (pOR) was significantly higher in individuals with hypertension[1.6(95%CI 1.24-2.00)], cardiovascular diseases [2.2 (95%CI 1.49- 3.27)], chronic obstructive pulmonary diseases [1.4(95% CI 1.05-2.00)] and diabetes [1.35(95%CI 1.06-1.73)].

**Conclusion:**

Our results indicates that the mortality in SARS-CoV-2 infected cancer patients is affected by preexisting non-cancer comorbidities. By identifying the comorbidities predictive for mortality, clinicians can better stratify the risk of cancer patients presenting with SARS-COV-2, on their initial contact with health services.

## Introduction

The outbreak of coronavirus disease also known as SARS CoV-2 in December 2019 has led to widespread impact globally and has been declared by World Health Organization (WHO) as a global public health emergency. Till August 25^th^ 2020, 2.3 million positive cases and 810,492 deaths globally have been attributed to COVID-19 (WHO, 2020). The highest burden of cases has been reported from United states, Brazil and India whereas highest deaths have been reported from the US, Brazil and United Kingdom (Worldometers.info, 2020). Novel coronavirus disease (SARS COV-2 infection or COVID-19) is a respiratory tract infection which has been linked with severe acute respiratory syndrome, is transmitted by human to human transfer of the virus through respiratory droplets, by direct contact with an infected person or through indirect contact with fomites in that environment (Li et al., 2020; Ong et al. 2020). Currently, more than 200 countries have been impacted by COVID-19. Most SARS-COV-2 patients develop mild to moderate symptoms, but patients in severe conditions present with acute respiratory distress syndrome (ARDS), multiple organ dysfunction syndrome (MODS) and septic shock (Cascella et al., 2020). Case fatality rate of SARS-COV-2 varies from 1.4% to 7.4% in different regions (Guan et al., 2020; Wang et al., 2020; Kahathuduwaet al., 2020). Mounting evidence has shown that patients with comorbidities are more likely to have severe disease sequel and mortality outcomes, as this virus induces abnormal and excessive non-effective host immune responses resulting in severe lung injury (Huang et al., 2020; Hui et al., 2020). SARS CoV-2 associated disease severity and outcomes have shown great variability with high predisposition among the elderly, those with underlying comorbidities and compromised immune system (Islam et al., 2020). It is shown that people with SARS CoV-2 infection are more likely to be elder, be obese and have underlying comorbidities including hypertension, diabetes, cardiovascular diseases (CVDs) and chronic respiratory diseases (COPD) (CDC, 2020a; Hu et al., 2020). Among patients with cancer, the pooled prevalence of COVID-19 has shown to be around 2% (95% CI: 2-3%) (Desai et al., 2020). In addition, variable case fatality rates have been shown by SARS-CoV-2 infected patients with different comorbidities including cancer (Sanyaolu et al. ,2020; Awadhesh et al., 2020). Cancer affects a significant portion of the population, with more than 18 million new cases per year globally (Bray et al., 2018). Though evidence is rapidly evolving of comorbidity burden including cancer and its impact on SARS CoV-2 outcomes, little information exists on burden of SARS CoV-2 infection by comorbidity burden among patients with preexisting cancer. Further limited evidence exists of impact of comorbidities among cancer patients on SARS CoV-2 outcomes. Being an important comorbid condition, cancer population has also shown increased rates of fatality when compared to non-cancer patients with SARS CoV-2 infection (Venkatesulu et al.,2020). Further as cancer patients tend to have an already compromised immune system due to either active disease or ongoing systemic therapy, it is possible that the SARS CoV-2 infection risks associated with underlying comorbidities among patients with pre-existing cancer might vary from those in non-cancer SARS CoV-2 infected patients. The relative impact of different underlying health conditions among patients with cancer and SARS CoV-2 infection remains yet to be explored. To address this gap, our review aims to explore prevalence of comorbidities among patients with pre-existing cancer and SARS CoV-2 infection and its impact on mortality.

## Materials and Methods

Our systematic review is registered with PROSPERO (CRD42020192901). We followed steps outlined for conducting systematic review by the Preferred Reporting Items for Systematic Reviews and Meta-Analyses (PRISMA) guidelines (Moher et al., 2009)

Search strategy- We performed our search on PubMed (Medline), Web of Science, Scopus and Embase with help of a health science librarian. We limited our search for articles published from in last one year (from 9th July 2019 till July 8th 2020). Keywords for our search included: SARS-COV-2 disease (COVID-19, 2019-nCoV, Novel coronavirus, coronavirus, SARS CoV-2) AND Cancer (Cancer, carcinoma, tumor, neoplasm, malignancy, oncology). In addition, a manual search of the reference lists was performed to look for relevant papers. 

Study selection criteria -All peer-reviewed and pre-print (not-peer-reviewed) studies met the pre-specified inclusion, and exclusion criteria were included in this study. These included:

Inclusion criteria(i) Patients having an existing diagnosis of cancer and are subsequently diagnosed with SARS-COV-2 infection by either: 1) dual fluorescence polymerase chain reaction (PCR) or quantitative real-time polymerase chain reaction (qRT-PCR), or 2) clinical diagnosis based on presentation (ii) presented prevalence of co-morbidities in SARS CoV-2 infected cancer patients (iii) presented outcomes including mortality rates following SARS-COV-2 among cancer patients with or without preexisting morbidity (iv) published in the English language and (v) studies with minimum sample size of 10. Studies that reported the prevalence of co-morbidities but did not report mortality by comorbidities were still included, but only incorporated in the pooled analysis of comorbidity prevalence. 

Exclusion criteria: Studies were excluded if SARS-COV-2 was reported among pregnant women or children (aged <18 years) and not written in English language, review papers, correspondence, viewpoints, editorials, commentaries, and studies where no information related to the previous morbidity was reported were also excluded.

*Data analysis*

*Data extraction (selection and coding)*

Two authors (HK and RP) independently screened all relevant titles and abstracts. All abstracts were coded as yes or no for full text screening. If both reviewers coded an abstract as “yes” it was included for full text screening, those which were coded as “no” by both screeners were excluded and if one was coded as “yes” or “no” were further evaluated for inclusion/exclusion by mutual discussion with a third reviewer (JST). The full texts of the articles were then retrieved and further screened, and then read by two reviewers (HK and RP) independently. Two authors used the pre-designed form to extract information independently. The following information was extracted: (1) study characteristics (first author, publication year, study setting, recruitment time frame), (2) population characteristics (age, sex, sample size), and outcomes of interest (total number of cancer patients with SARS CoV-2 infection, comorbidity burden among cancer patients, mortality rates among patients with cancer and by comorbidity type if available) Disagreements reported in data extraction were reviewed and solved by the corresponding author (JST). 

*Quality assessment*

Newcastle Ottawa Scale (NOS) for observational studies was used to evaluate the quality of the studies included in the review.Both authors (HK and RP) read all papers and assessed the methodological quality and scoring was done independently. Any disagreements were resolved through discussion. We performed quality assessment on the included studies. This scale assesses quality of included studies on three groups: Selection, Comparability, and Assessment. Reviewers rate studies on scale of 0–4 for selection, scale of 0–2 for comparability, and scale of 0–3 for ascertainment respectively (Wells et al., 2000; Taylor et al., 2020). Funnel plots and egger’s test were utilized to assess for publication bias (Thomas, 1998)

*Statistical analysis*

Data analyses was performed by Microsoft Excel and Stata version 15 software (StataCorp, Texas, USA)(Stata Corp.,2017). Pre-existing comorbidities among SARS-COV-2 infected cancer patients reported in the selected studies were grouped into six broad categories including hypertension, cardiovascular disease, chronic obstructive pulmonary disease , diabetes, chronic kidney disease and cerebrovascular diseases. We calculated the estimated pooled prevalence of these diseases among SARS CoV-2 infected cancer patients. We also calculated the estimated pooled risk of mortality by comorbidity burden among cancer patients with SARS CoV-2 infection .Random effects models were used to account for effect estimate which was reported as odds ratios (ORs) and its 95% confidence intervals (CIs). Two tailed-value set at 0.05 statistical significance was used. Heterogeneity between studies was estimated with Tau-square test. Further forest plots were plotted to display mean effect estimates and funnel plots were plotted to assess for heterogeneity. 

## Results

**Figure 1:**
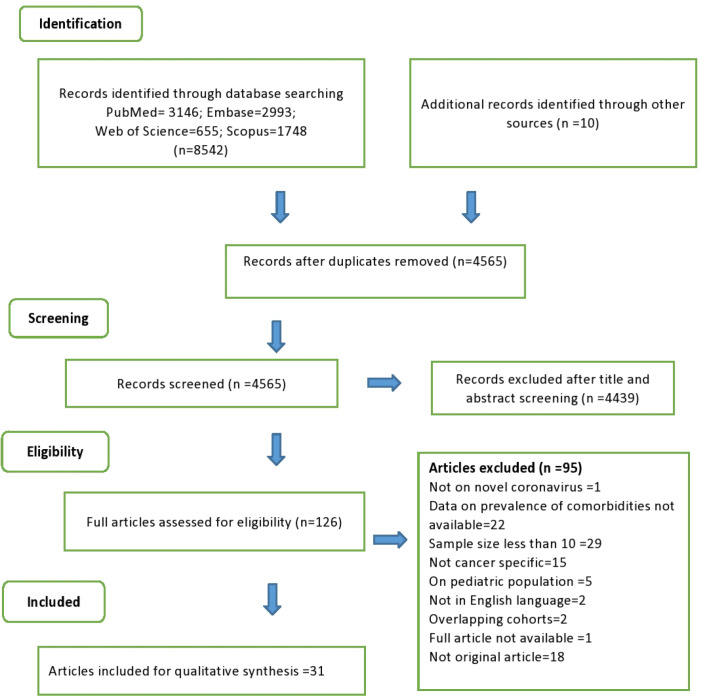
Overview of Our Selection Process (PRISMA flow Chart)

Our literature search retrieved 8,923 articles. After removing of duplicates, 4,565 articles were screened, of which 126 articles underwent full review. Further 95 studies were excluded and 31 were included in the final analysis. Figure 1 shows an overview of our selection process (PRISMA flow Chart).

Table 1 provides an overview of study characteristics including population characteristics (including total number of study subjects, age, sex and outcome i.e. mortality associated with SARS CoV-2 infection).The included studies were from China (n = 9) (Dai et al., 2020; He et al., 2020; Meng et al., 2020; Wang et al., 2020; Yang et al., 2020; Yang et al., 2020; Zhang et al., 2020; Zhang H et al., 2020; Tian et al.,2020), the United States (n = 5) (Kalinsky et al., 2020; Luo et al., 2020; Mehta et al., 2020; Robilotti et al., 2020; Wang et al., 2020), Italy (n = 4) (Bogani et al., 2020; Fattizzo et al., 2020; Gallo et al., 2020; Stroppa et al., 2020), Spain (n = 2) (Rogado et al., 2020; Yarza et al., 2020), the United Kingdom (n =5) (Aries et al., 2020; Russell et al. 2020; Shah et al., 2020; Shi et al., 2020; Lee et al., 2020), France (n = 2) (Basse et al., 2020; Vuagnat et al., 2020), Brazil (n=1)( de Melo et al., 2020), Belgium (n = 1)( Lattenist et al., 2020) , Russia (n=1) ( Moiseev et al., 2020) and one study from Europe included patients from 8 countries (Garassino et al., 2020). Recruitment in these 18 studies was from December 17^th^ 2019 to June17^th^ 2020. A total of 4086 cancer cases infected with SARS-CoV-2 with sample size of studies varying between 13 to 800 were included. The median age of our participants ranged from 35 to 77 years. Proportion of men across these studies from 0 to 77%. Majority of participants in the included studies were males, except for two which included only women with gynecological cancers. Among patients with SARS CoV-2 infection and cancer, overall mortality rates ranged from ranged from 3.7% to 61.5%. Highest mortality in hematological cancers was reported by stroppa et al., (2020) (100%) and He et al., (2020) (61.5%). Among solid tumors highest mortality was reported by stroppa et al., (2020) in breast carcinoma (100%) and Yang et al., (2020) in prostate cancer patients(100%). Mehta et al., (2020) reported 67% mortality in pancreatic cancer atients. Among lung cancer patients highest mortality(55%) was reported by Mehta et al.,(2020). When segregated by regions, mortality ranged from 11.4% to 61.5% in China, 15.8% to 36% in Italy, 25.4% to 42.2% in Spain, 8% to 17% in France,18% to 40.% in the United Kingdom, 3.7% to 28%. in the United States. Studies from Brazil and Belgium reported 33.1% and 46% mortality respectively. Another study done in eight countries of Europe reported mortality rate as 33%.

**Figure 2:**
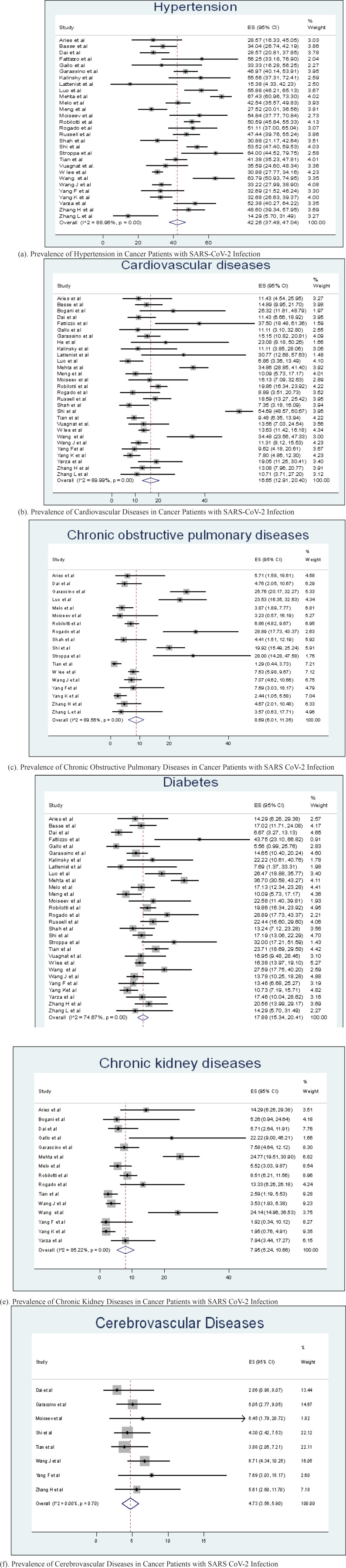
Forest Plots for Meta-Analyses of Prevalence of Co-Morbidities

Our analysis identified that the pooled prevalence of comorbidities among patients with SARS CoV-2 infection and cancer were as follows: hypertension: 42.3% (95%CI:37.5- 47.0; p <0.0001), cardiovascular diseases: 16.7% (95%CI:12.9-20.4; p <0.0001), chronic obstructive pulmonary diseases : 8.69% (95% CI: 6.01-11.36; p <0.0001), diabetes:17.8% (95% CI: 15.3-20.4; p <0.0001), chronic kidney disease: 8.0% (95%CI:5.24-10.66; p <0.0001 and cerebrovascular disease:4.7% (95% CI:3.56- 5.90; p <0.0001) respectively. Figure 1S(a-f) provides an overview of our pooled analysis using funnel plots. Table 2 provides a summary of our pooled analysis along with results of heterogeneity which ranged from low for cerebrovascular diseases to high for other diseases including hypertension, cardiovascular diseases, chronic obstructive pulmonary diseases and chronic kidney diseases with full forest plots provided in Figure 2 (a-f). Few studies which have not specified COPD and chronic kidney diseases as a separate group of diseases were included in narrative review along with other diseases (Table S1).

The pooled ORs of deaths for each category of pre-existing morbidities among SARS CoV-2 infected cancer patients are presented in Table 2.

**Figure 3:**
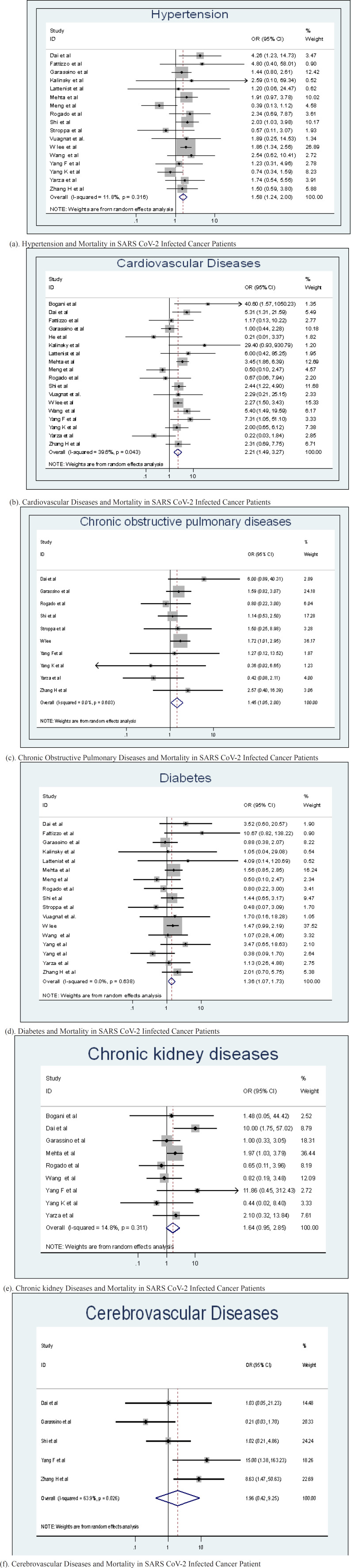
Meta-Analyses of Mortality in SARS CoV-2 Infected Cancer Patients by Co-Morbidity

Hypertension: Seventeen included studies reported the relationship between hypertension and mortality in cancer patients with SARS CoV-2 infection. Pooled analysis showed that presence of hypertension was an important risk factor for mortality among cancer patients. (OR: 1.6, 95% CI: 1.24-2.00, p=0.000, I^2^ =11.8%, pheterogeneity= 0.316) among these studies. Between-study variance was not found to be significant (Tau-squared = 0.0281). Pooled effects of hypertension on the mortality linked with SARS CoV-2 infection in cancer patients are shown in Figure 3(a). 

Cardiovascular diseases: Combining the reported estimates from 18 studies, we found a significant positive association between cardiovascular diseases (OR: 2.2, 95% CIs :  1.49-3.27, p  =0.000, I2=39.6%, pheterogeneity=0.043) and risk of death from SARS CoV-2 infection. Between-study variance was found to be significant (Tau-squared = 0.216). Pooled effects of cardiovascular diseases on the mortality associated with SARS CoV-2 infection in cancer patients are shown in Fig.3(b). Cardiovascular diseases includes patients with coronary artery diseases, myocardial infarction, ischemic heart disease, congestive heart failure (either or all of these). 

Chronic obstructive pulmonary diseases: The pooled analysis of ten studies, shows significant association between mortality and chronic obstructive pulmonary diseases in SARS CoV-2 infected cancer patient (OR= 1.4, 95% CI:1.05-2.00, p=0.025, I^2^ =0, pheterogeneity= 0.603, Tau-squared = 0.00) as shown in Fig 3 (c). There were about five more studies which were not included in meta-analysis as they have not reported chronic obstructive pulmonary diseases separately. These studies are included in narrative review (Table S1).

Diabetes : Association between diabetes and mortality in SARS CoV-2 infected cancer patients by reported seventeen studies, was found to be significant (OR =1.35, 95% CI:1.06-1.73 , p=0.013 , I^2^ =00%, pheterogeneity= 0.638, Tau-squared = 0.00 ) as shown in Figure 3(d).

Chronic kidney diseases: The meta-analysis of nine studies shows statistically non-significant association between chronic kidney diseases with an overall higher risk of mortality among patients with SARS CoV-2 infection and cancer (OR: 1.64, 95% CI: 0.95-2.84, p= 0.077, I^2^ =14.83%, pheterogeneity= 0.311, Tau-squared = 0.1046). Figure 3(e). There were few more studies which reported data on renal diseases, but not specified it as chronic kidney diseases were not included in meta-analysis but reported in narrative review (Table S1).

Cerebrovascular diseases – Data reported by five studies included in meta-analysis shows that the association between cerebrovascular diseases with the mortality in SA RS CoV-2 infected cancer patients is non-significant. (OR: 1.96 , 95% CI: 0.42-9.25, p=0.393, I^2^ =63.9%, pheterogeneity= = 0.026, Tau-squared = 1.9418) Figure3(f).

The risk of publication bias was found to be non-significant on applying eggers test.(Table 2; the funnel plots are provided in Figure 1S). Table 3. provides an overview of the quality assessment of our included studies. Of 18 included studies, we found 21 to be of high quality, 5 to be medium quality and 5 to be of low quality based on their average scores on the Newcastle Ottawa Scale.

**Table 1:** Characteristics of Included Studies in Systematic Review

Author	Study location	Study period	Study design	Sample Size	Median Age in years(IQR	Gender Male (%)	Mortality	Hyper-tension	Cardiovas-cular diseases	Chronic respiratory diseases	Diabetes	Chronic kidney diseases	Cerebrovascular diseases
							N (%)	N (%)	N (%)	N (%)	N (%)	N (%)	N (%)
Aries et al	UK	March 11,2020 to May 11, 2020	Retrospective case series	35	69(31–87)*	66	14(40)	10 (28.57)	4(11.43)	4(11.43)	5(14.29)	5(14.29)	NR
Basse et al	France	March 13, 2020, to April 25,	Prospective cohort study	141	62 (52-72)	28	26(18)	48(34.04)	21(14.89)	7(4.96)	24(17.02)	NR	NR
Bogani et al	Italy	February to March 2020	Retrospective case series	19	65 (49- 84)*	0% (all females)	3(15.8)	NR	5(26.32)	NR	NR	1(5.26)	NR
Dai et al	China	January 1,2020 to February 24, 2020.	Retrospective case series	105	64 (50-78)	57	12(11.4)	30(28.57)	12(11.43)	6(5.71)	7(6.67)	6(5.71)	2(1.90)
Fattizzo et al	Italy	March 10, 2020 to April 24, 2020	Retrospective case series	16	77(27–94)*	62.5	5(31.3)	9(56.25)	6(37.50)	1(6.25)	7(43.75)	2(12.50)	NR
Gallo et al	Italy	February 29,2020 to April 11, 2020.	Retrospective cohort study	18	73.7**	66.7	6(33.3)	6(33.33)	2(11.11)	2(11.11)	1(5.56)	4(22.22)	NR
Garassino et al	Europe	March 26, 2020 and April 12, 2020	Retrospective cohort study	198	68 (61·8–75)	70%	66(33)	93(46.97)	30(15.15)	51(25.76)	29(14.65)	15(7.58)	10(5.05)
He et al	China	January 23, 2020 to February 14, 2020	Retrospective cohort study	13	35 (23 -53)	53.8	8 (61.5)	NR	3(23.08)	NR	NR	NR	NR
Kalinsky et al	USA	March 10, 2020 to April 29, 2020	Retrospective case series	27	56 (32–87)*	3.7	1(3.7)	15(55.56)	3(11.11)	6(22.22)	6(22.22)	NR	NR
Lattenist et al	Belgium	March 13,2020 to May 15, 2020	Retrospective case series	13	70 (59–79)	77	6(46%)	2(15.38)	4(31)	3(23.08)	1(7.69)	3(23.08)	NR
Luo et al	USA	March 12, 2020 to May 6, 2020		102	68 (61-75)	48	25(25%)	57(55.88)	7(6.86)	52(50.98)	27(26.47)	NR	NR
Mehta et al	USA	March 18 2020 to April 8 2020	Retrospective case series	218	69(10-92)*	58	61(27.99)	147(67.43)	76(34.86)	62(28.44)	80(36.70)	54(24.77)	NR
Melo et al	Brazil	April 30, 2020 to May 26, 2020	Retrospective cohort study	181	55.3 (SD±21.1)	39.2	60 (33.1)	77(42.54)	NR	7(3.87)	31(17.13)	10(5.52)	NR
Meng et al	China	January 18,2020 and March 27, 2020,	Retrospective case series	109	61.7 (45.6-78)	56	32(29.4)	30(27.52)	11(10.09)	NR	11(10.09)	NR	NR
Moiseev et al	Russia.	Not reported	Retrospective cohort study	31	66**	51.6	Not reported	17(54.84)	5(16.13)	2(6.45)	7(22.58)	NR	2(6.45)
Robilotti et al	USA	10 March,2020 to May 7, 2020	Retrospective cohort study	423	56***	50	51(12)	214(50.59)	84(19.86)	72(17.02)	84(19.86)	36(8.51)	NR
Rogado et al	Spain	February 1, 2020, to April 7, 2020	Retrospective case series	45	71(34-90)*	66.7	19(42.2)	23(51.11)	4(8.89)	13(28.89)	13(28.89)	3(6.67)	NR
Russell et al	UK	February 29,2020 to May 12, 020.	Retrospective cohort study	156	65.18 (SD:14.8)	57.7	34(22)	74(47.44)	29(18.59)	25(16.03)	35(22.44)	30(19.23)	NR
Shah et al	UK	March 13, 2020 to April 15, 2020		68	73 (62-82)	67.6	39* CDR	21(30.88)	5(7.35)	8(11.76)	9(13.24)	NR	NR
Author	Study location	Study period	Study design	Sample Size	Median Age in years(IQR	Gender Male (%)	Mortality	Hyper-tension	Cardiovas-cular diseases	Chronic respiratory diseases	Diabetes	Chronic kidney diseases	Cerebrovascular diseases
							N (%)	N (%)	N (%)	N (%)	N (%)	N (%)	N (%)
Shi et al	UK	till June 17th , 2020	Prospective cohort study	256	61.6 (55.5-8.5)	54.1	46(18)	137(53.52)	140(54.69)	77(30.08)	44(17.19)	NR	11(4.30)
Stroppa et al	Italy	February 21, 2020 to March 18,2020	Retrospective case series	25	71.64 (50–84 )*	80	9(36)	16(64)	NR	7(28)	8(32)	NR	NR
Tian et al	China	January 13, 2020 to March 18, 2020	Retrospective cohort study	232	64·0 (58–69)	51	46(20)	96(41.38)	22(9.48)	3(1.29)	55(23.71)	6(2.59)	9(3.88)
Vuagnat et al.	France	March 13, 2020, to April 25, 2020	Prospective cohort study	59	58 (48–68)	0% (all females)	4(7)	21(35.59)	8(13.56)	2(3.39)	10(16.96)	NR	NR
W lee et al	UK	March 18, 2020 to April 26, 2020	Prospective cohort study	800	69 (59–76)	56	226(28.3)	247(30.88)	109(13.63)	61(7.63)	131(16.38)	NR	NR
Wang B et al	USA	March 1, 2020 and April 30, 2020	Retrospective case series	58	67 (54.5-79.5)	52	14(24)	37(63.79)	20(34.48)	12(20.69)	16(27.59)	14(24.14)	NR
Wang J et al	China	December 17, 2019 to March 18, 2020	Retrospective case series	283	63 (55-70)	50	47(18)	94(33.22)	32(11.31)	20(7.07)	39(13.78)	10(3.53)	7(2.47)
Yang F et al	China	January 11, 2020 to April 15, 2020	Retrospective case series	52	63(34-98)*	53.8	11(21.2)	17(32.69)	5(9.62)	4(7.69)	7(13.46)	1(1.92)	4(7.69)
Yang K et al	China	January 13, 2020 to March 18, 2020	Retrospective cohort study	205	63 (56–70)	47	40(19.5)	67(32.68)	16(7.80)	5(2.44)	22(10.7)	4(1.95)	NR
Yarza et al	Spain.	March 9, 2020 to April 19, 2020	Retrospective case series	63	66(63.4-68.8)***	54%	16(25.4)	33(52.38)	12(19.05)	14(22.22)	11(17.56)	5(7.94)	NR
Zhang H et al	China	January 5, 2020 to March 18, 2020	Retrospective cohort study	107	66(36-98)*	56.1	23 (21.5%)	52(48.60)	14(13.08)	5(4.67)	22(20.56)	NR	6(5.61)
Zhang L et al	China	January 13, 2020 to February 26, 2020.	Retrospective case study	28	65 (56-70)	60.7	8 (28.6)	4(14.29)	3(10.71)	1(3.57)	4(14.29)	NR	NR

*, Age in years (range); **, Range or IQR not reported; ***, Mean age; ****, 56% patients were above 60 years of age; median age not reported; SD, (Standard deviation); ,IQR, interquartile range; NR, Not reported

**Table 2:** Summary of Meta-Analyses Results for Prevalence of Co-Morbidities and Risk of Mortality by Comorbidities, in SARS CoV-2 Infected Cancer Patients

Comorbidities	No. of studies	Pooled effect size(95%CI), p-value	I^2^(%), P-value	Tau^2	Egger's (P-value)
Estimated pooled prevalence (%) of co-morbidities in cancer patients with SARS CoV-2 infection					
Hypertension	29	42.3% (95%CI 37.5- 47.0), 0.00	88.96%, 0.00	0.01	0.439
Cardiovascular diseases	29	16.7% (95%CI 12.9-20.4),0.00	89.99%,0.00	0.01	0.252
Chronic obstructive pulmonary diseases	18	8.69% (95% CI 6.01-11.36),0.00	89.56%,0.00	0	0.008
Diabetes	29	17.8% (95% CI 15.34-20.41),0.00	74.67%,0.00	0	0.174
Chronic kidney diseases	15	8.0% (95%CI 5.24-10.66),0.00	85.22% 0.00	0	0.007
Cerebrovascular diseases.	8	4.73 % (95% CI 3.56 -5.90),0.00	0.00%, 0.70	0	0.259
Estimated pooled OR of mortality from SARS CoV-2 infected cancer patients if they have a co-morbidity compared with if they do not					
Hypertension	17	1.6(95%CI 1.24-2.00), 0.000	11.8%, 0.316	0.0281	0.723
Cardiovascular diseases	18	2.2 (95%CI 1.49- 3.27),0.000	39.6%, 0.043	0.216	0.903
Chronic obstructive pulmonary diseases	10	1.4(95% CI 1.05-2.00), 0.025	0.0%, 0.603	0	
Diabetes	17	1.35(95%CI 1.06-1.73), 0.013	0.0%, 0.638	0	0.902
Chronic kidney diseases	9	1.64(95%CI 0.95-2.84), 0.077	14.8%, 0.311	0.1046	0.006
Cerebrovascular diseases.	5	1.96(95% CI 0.42-9.25), 0.393	63.9%, 0.026	1.9418	0.956

**Table 3:** Quality Assessment of the Included Observational Studies by NOS

Sr. No.	Domain	SELECTION				COMPARABILITY		OUTCOME			
	Study	Representat-iveness of the exposed cohort	Selection of the non exposed cohort	Ascertain-ment of exposure	Demonstration that outcome of interest was not present at start of study	Comparability of cohorts on the basis of the design or analysis		Assessment of outcome	Was follow-up long enough for outcomes to occur	Adequacy of follow up of cohorts	Overall score
1	Aries et al	*	*	*	*			*	*	*	7
2	Basse et al	*		*	*	*	*	*	*	*	8
3	Bogani et al	*	*	*	*			*	*	*	7
4	Dai et al	*	*	*	*	*	*	*	*		8
5	Fattizzo et al	*	*	*	*			*			5
6	Gallo et al	*	*	*	*	*	*	*			7
7	Garassino et al	*	*	*	*	*		*	*	*	8
8	He et al	*	*	*	*			*		*	7
9	Kalinsky et al	*		*	*			*	*	*	6
10	Lattenist et al	*	*	*	*			*		*	7
11	Luo et al	*	*	*	*	*		*			6
12	Mehta et al	*	*	*	*	*		*			6
13	Melo et al	*	*	*	*		*	*		*	7
14	Meng et al	*	*	*	*	*		*		*	7
15	Moiseev et al	*		*	*			*			4
16	Robilotti et al	*		*	*	*	*	*	*	*	8
17	Rogado et al	*	*	*	*	*		*		*	7
18	Russell et al	*	*	*	*	*		*		*	7
19	Shah et al	*	*	*	*	*		*	*	*	8
20	Shi et al	*	*	*	*			*			5
21	Stroppa et al	*	*	*	*	*		*		*	7
22	Tian et al	*	*	*	*	*		*		*	7
23	Vuagnat et al.	*		*	*			*	*		5
24	W lee et al	*	*	*	*	*	*	*			7
25	Wang et al	*		*	*	*	*	*	*	*	8
26	Wang J et al	*	*	*	*	*		*	*	*	8
27	Yang F et al	*	*	*	*			*	*	*	7
28	Yang K et al	*	*	*	*			*	*	*	7
29	Yarza et al	*		*	*			*		*	5
30	Zhang H et al	*	*	*	*			*		*	6
31	Zhang L et al	*		*	*	*		*		*	6

(a). Prevalence of Hypertension in Cancer Patients with SARS-CoV-2 Infection

(b). Prevalence of Cardiovascular Diseases in Cancer Patients with SARS-CoV-2 Infection

(c). Prevalence of Chronic Obstructive Pulmonary Diseases in Cancer Patients with SARS CoV-2 Infection

(d). Prevalence of Diabetes in Cancer Patients with SARS CoV-2 Infection

(e). Prevalence of Chronic Kidney Diseases in Cancer Patients with SARS CoV-2 Infection

(f). Prevalence of Cerebrovascular Diseases in Cancer Patients with SARS CoV-2 Infection

(a). Hypertension and Mortality in SARS CoV-2 Infected Cancer Patients

(b). Cardiovascular Diseases and Mortality in SARS CoV-2 Infected Cancer Patients

(c). Chronic Obstructive Pulmonary Diseases and Mortality in SARS CoV-2 Infected Cancer Patients

(d). Diabetes and Mortality in SARS CoV-2 Iinfected Cancer Patients

(e). Chronic kidney Diseases and Mortality in SARS CoV-2 Infected Cancer Patients

(f). Cerebrovascular Diseases and Mortality in SARS CoV-2 Infected Cancer Patient

## Discussion

Our study remains one of the first to systematically quantify the burden of comorbidity among cancer patients impacted with SARS CoV-2 infection and their impact on mortality associated with SARS CoV-2 infection. We found that in addition to having high rates of mortality among cancer patients, presence of comorbidities including hypertension, cardiovascular diseases, COPD and diabetes were also associated with poor overall mortality. Hence it remains crucial to consider burden of multimorbidity among cancer patients with SARS CoV-2 infection and develop prevention and treatment strategies.

Prior studies on estimates of burden of cancer among SARS CoV-2 infection patients has shown to be around 2% (Gao et al., 2020).This highlights the greater challenge public health and clinical entities face as more and more patients are diagnosed with SARS CoV-2 infections increase among those with preexisting conditions including cancer. Additionally, case fatality rates in cancer patients have been reported to be twice as compared to the general population with SARS-CoV-2 infection. Cancer patients are more substile to have severe outcomes due to SARS-CoV-2 infection because of other associated factors including old age, multiple preexisting comorbidities, ongoing active therapy and associated side effects and an overall immunocomprised state, so they should have priority access to services (Basu, 2020). Further, owing to oncologic interventions and follow-up thereof, they spend more time in the hospital and with healthcare providers which may further increase their exposure to develop infections. This points towards the need to devise precise strategies to deal with specific cancer populations during pandemic times (Mousavi et al., 2020; Dewi et al., 2020)

We found that in addition to preexisting conditions, prevalence of SARS CoV-2 infections were more common among men, among older adults with cancer. Comorbid conditions are likely to decrease immunity, impair macrophage and lymphocyte function, and are associated to the pathogenesis of SARS-CoV-2 (Landi et al., 2020). Mortality rates reported by the studies included in meta-analysis varies from 3.7% to 63.1 % in breast cancer and hematological cancer patients respectively.

Previous reports and systematic review of observational studies shows that most frequently reported co-morbidities were hypertension (16-23%), cardiovascular disease (5-16.4%), diabetes (8-11.5%), cancer (3.9%), chronic kidney disease (2.4%), chronic obstructive pulmonary diseases (3.1%) and cerebrovascular diseases (3%) among patients with SARS CoV-2 (Awadhesh et al., 2020; Emami et al., 2020; Yang J et al.,2020; Li et al., 2020).

Among cancer patients most prevalent comorbidities reported are hypertension (46.6%), diabetes mellitus (20.4%), cardiac disease (34.9%), cerebrovascular disease (9.1%), chronic liver disease (9.2 %), chronic kidney disease (10.8%) and chronic lung disease (14.7%) (Venkatesulu et al., 2020).

According to current analysis, hypertension (42.3%), cardiovascular disease (16.7%), diabetes (17.8%), chronic kidney disease (8%), chronic obstructive pulmonary diseases (8.7%) and cerebrovascular diseases (4.7%) were most prevalent comorbidities among cancer patients with SARS CoV-2 infection.

Further, hypertension, cardiovascular disease, diabetes and chronic obstructive pulmonary diseases were associated with an increased risk of mortality in cancer patients with SARS CoV-2 infection. SARS Cov2 infected cancer patients with cardiovascular diseases had a 2.2 fold higher risk of progression than patients without cardiovascular diseases. Comorbidities such as, cardiovascular diseases or renal diseases leads to increase in abrupt loss of kidney function, tissue damage that causes hypoxia, shock, and rhabdomyolysis, and increased occurrence of thrombocytopenia (reduced platelet counts) (Cheng et al., 2020; Zhou et al., 2020; Vanmassenhove et al., 2017; Lau et al., 2017; Tisoncik et al., 2012; Boettler et al., 2020). These could independently elevate the risk of death and add to the adverse effects on the human body being infected with SARS-COV-2. 

Patients with chronic respiratory disorders like chronic obstructive pulmonary disease, are prone to developing ARDS because of their lower resistance to the virus. We have identified significantly increased risk of mortality in individuals with chronic obstructive pulmonary diseases. In diabetic patients it induces inflammatory infection by causing the accumulation of activated innate immune cells in metabolic tissues which leads to the release of inflammatory mediators, especially IL-1β and TNFα (Odegaard et al., 2012). Our meta-analysis also indicates that there was significant association between diabetes and SARS CoV-2 infected cancer patients’ increased risk of mortality. However, we did not find a significant association between chronic kidney diseases and cerebrovascular diseases with risk of mortality in cancer patients with SARS CoV-2 infection. Hence given the high risk associated with comorbidities, patients with these condition and cancer likely suffer from a heighted weakened immune system, a greater burden of treatment associated complications, multipharmacy and clinic visits and heightened exposure to clinic and hospital setting. 

In comparison to general population, prevalence of comorbidities among patients with cancer including hypertension, cardiovascular diseases, chronic respiratory diseases, chronic kidney diseases and cerebrovascular diseases was found to be higher except for diabetes (Deasi et al., 2020; Yang et al., 2020). Further mortality risk associated with hypertension, cardiovascular diseases, diabetes, chronic obstructive pulmonary diseases, chronic kidney diseases and cerebrovascular disease was reported to be significantly higher in general population (Huang et al., 2020; Desai et al., 2020). Similarly, in cancer patients risk of mortality is significantly higher in presence of hypertension, cardiovascular diseases, diabetes, chronic respiratory diseases while mortality risk due to chronic kidney diseases and cerebrovascular diseases was found to be non-significant, as shown by the results of our meta-analysis. 

Our study has several strengths and limitations. First, the sample size across studies varied which might impact our overall estimates. Follow-up of patients in reported by different studies was found to be variable. We were not able to identify or take into account treatment strategies for these patients, as their current disease status might also impact outcomes. Although we have tried to exclude the data from overlapping cohorts, it was not possible to identify every possible overlap among all the studies. As many of these chronic conditions may be co-existing, which would have been a useful factor to assess, but due to paucity of data we were unable to assess aspects of multimorbidity among patients. Further, as burden of cancer and comorbidities varies across geographical regions, our estimates might have limited generalizability to other regions from where limited to no data exists. Other risk factors like age, recent history of anticancer treatment, and stage of cancer could also significantly impact the overall mortality, were not analyzed in the present meta-analysis, however results on stage and type of anticancer treatment reported by the included studies are given in narrative review (Table S1).

Our strengths include the use of robust methods, with a comprehensive search strategy of multiple databases, with the use of all available data including grey literature to estimate burden of comorbidities among cancer patients. Further, we pooled estimates across studies from different regions to give a holistic picture of the rapidly evolving pandemic

Our review concludes that most SARS Cov-2 infected cancer patients have non-cancer comorbid conditions and these comorbidities confer additional mortality risk. We have found that risk of death was found to be higher among SARS-COV-2 patients who had comorbidities like hypertension, cardiovascular diseases, chronic obstructive pulmonary diseases and diabetes. Management of cancer patients with underlying comorbidities remains crucial in the era of pandemic. 

## Author Contribution Statement

HK and JST conceived the idea for the study. HK and RP carried out the literature search, screened the studies, and undertook data extraction. HK & RP carried out the statistical analysis. HK and RP carried out the risk of bias scoring. HK and SA interpreted the findings; HK drafted the manuscript, and produced the figures. HK, RP, JST and SA critically reviewed the manuscript. HK and SA revised the manuscript for final submission. All authors approved the final version of the manuscript
